# Supplementary material for: Polarization bandgaps and fluid-like elasticity in fully solid elastic metamaterials
Source: Nat Commun. 2016 Nov 21;7:13536. doi: 10.1038/ncomms13536 (PMC5121349; doi:10.1038/ncomms13536)
Supplement: Supplementary Information — Supplementary Figures 1-9, Supplementary Notes 1-7 and Supplementary References [file ncomms13536-s1.pdf]

SUPPLEMENTARY FIGURES

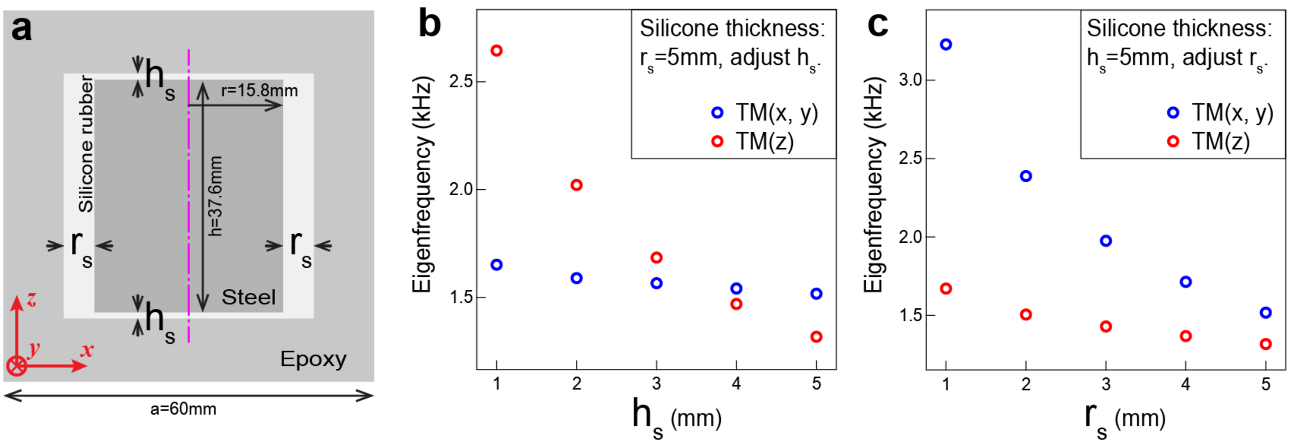

Supplementary Figure 1 Tuning the eigenfrequencies of the translational modes. A cutaway drawing of the metamaterial unit cell is shown in **a**. Here, h_s denotes the silicone rubber's thickness on the top and at the bottom of the steel cylinder, and r_s denotes the silicone rubber's thickness around the side surface. **b** shows the dependence of the eigenfrequencies of translational modes (TMs) on h_s, with r_s fixed at 5mm. **c** shows the dependence of the eigenfrequencies of TMs on r_s, with h_s fixed at 5mm. It is seen that TM(*x, y*) and TM(*z*) are separately tunable.

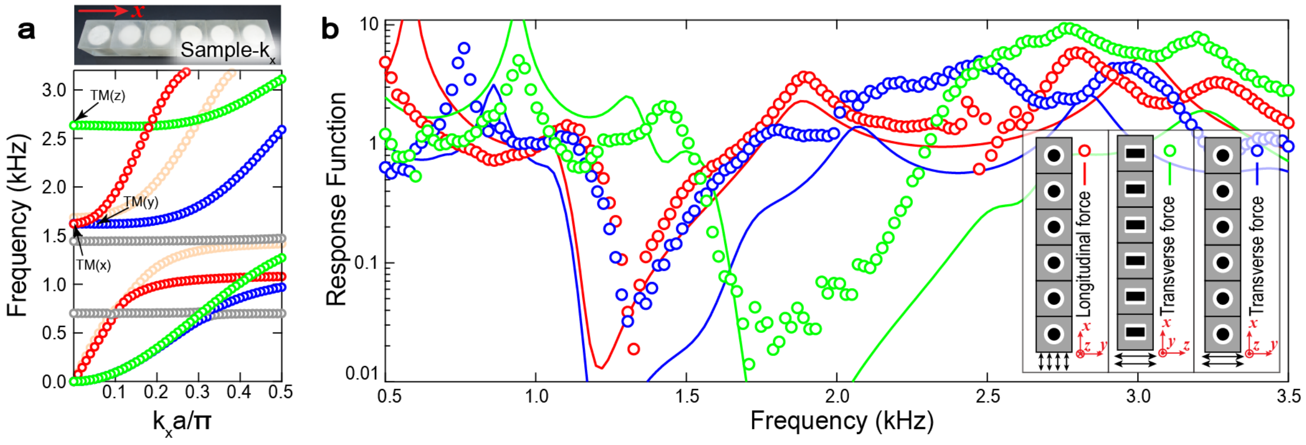

Supplementary Figure 2 Transverse and longitudinal response of sample-k_x. A photographic image of sample-k_x is shown in **a**. Band structure of sample-k_x is also shown in **a**. Here, red markers represent the longitudinal branch and TM(*z*); blue/green makers represent the flexural branches and TM(*x*)/TM(*y*), respectively. Orange markers show the torsional branch and rotational modes with about *x*-axis (RM(*x*)), which are already discussed in the main text and therefore are dimmed here. Grey markers delineate RM(*y*) and RM(*z*) which do not couple to external stimulations, therefore manifest as flat bands. **b** shows the measured (markers) and simulated (curves) response functions of sample-k_x. Sample-k_x is partially fluid-like in 1.7–2.6 kHz, in which the *z*-polarized flexural waves (green) cannot propagate, whereas longitudinal (red) and *y*-polarized (blue)

flexural waves are both allowed.

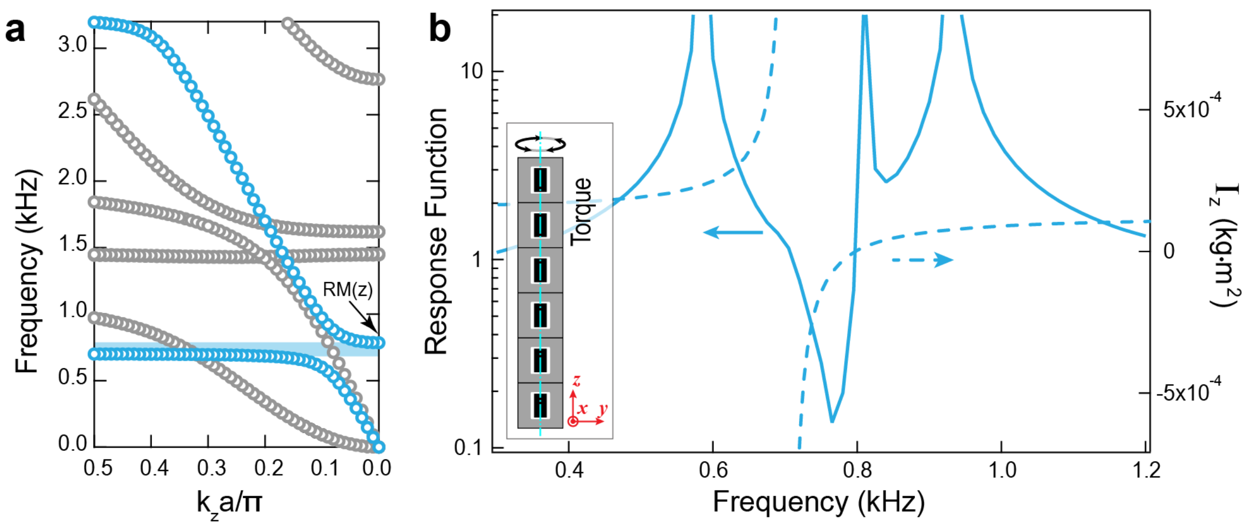

Supplementary Figure 3 Torsional response of sample-k_z. The band structure of sample-k_z is shown in **a**, in which the torsional branch and rotation mode (RM(*z*)) are highlighted in cyan. The cyan shaded region is a torsional bandgap. The numerically obtained response function of sample-k_z under rotational excitation is shown in **b** (solid curves, left axis). Effective moment of inertia about *z*-axis (denoted I_z) is plotted as a function of frequency (dashed curves, right axis).

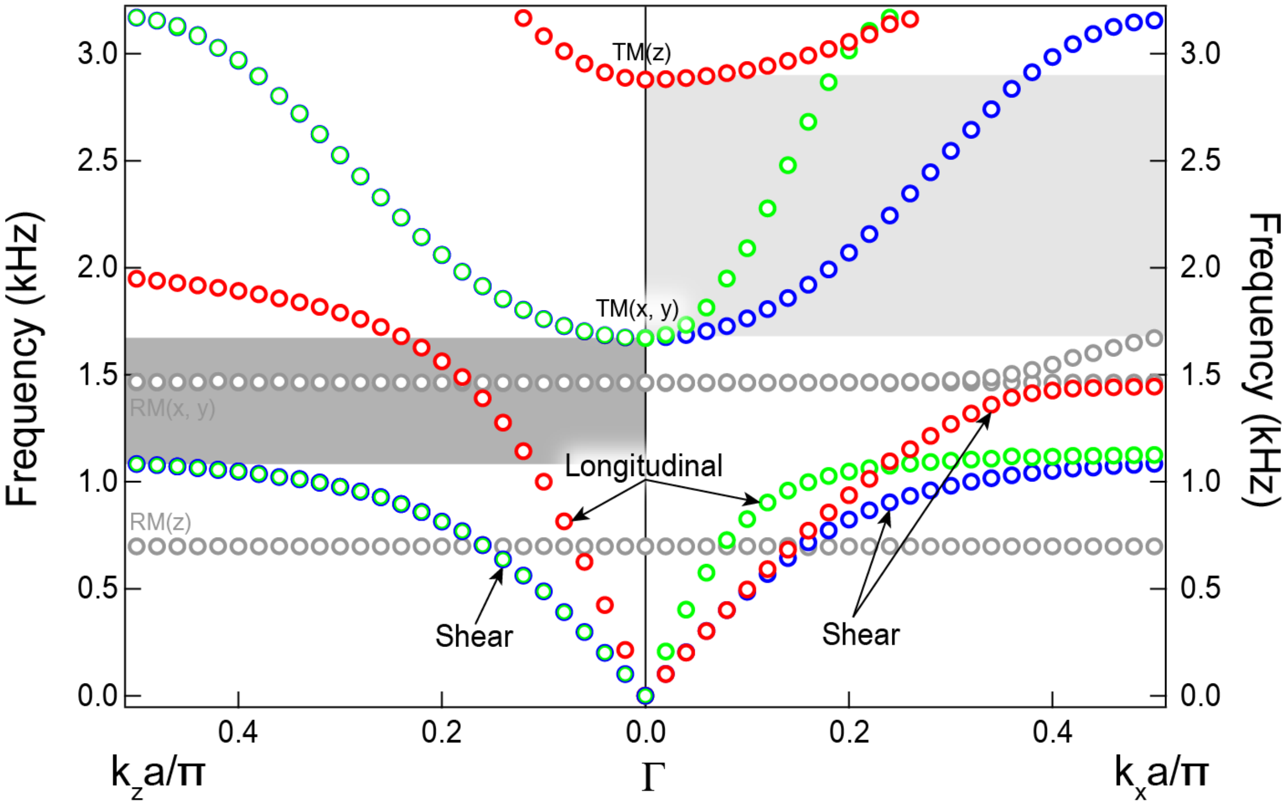

Supplementary Figure 4 Band structure of the three-dimensional lattice. Branches that couple with TM(*z*)

/ TM(*x*, *y*) are in red / blue (green), respectively. Fluid-like property is found for waves that propagate in *z* direction, which is shaded dark grey (~1.1–1.7 kHz). Light grey region (~1.7–2.9 kHz) is partially fluid-like: *z*-polarized shear waves cannot propagate, but both longitudinal and *y*-polarized shear waves are allowed.

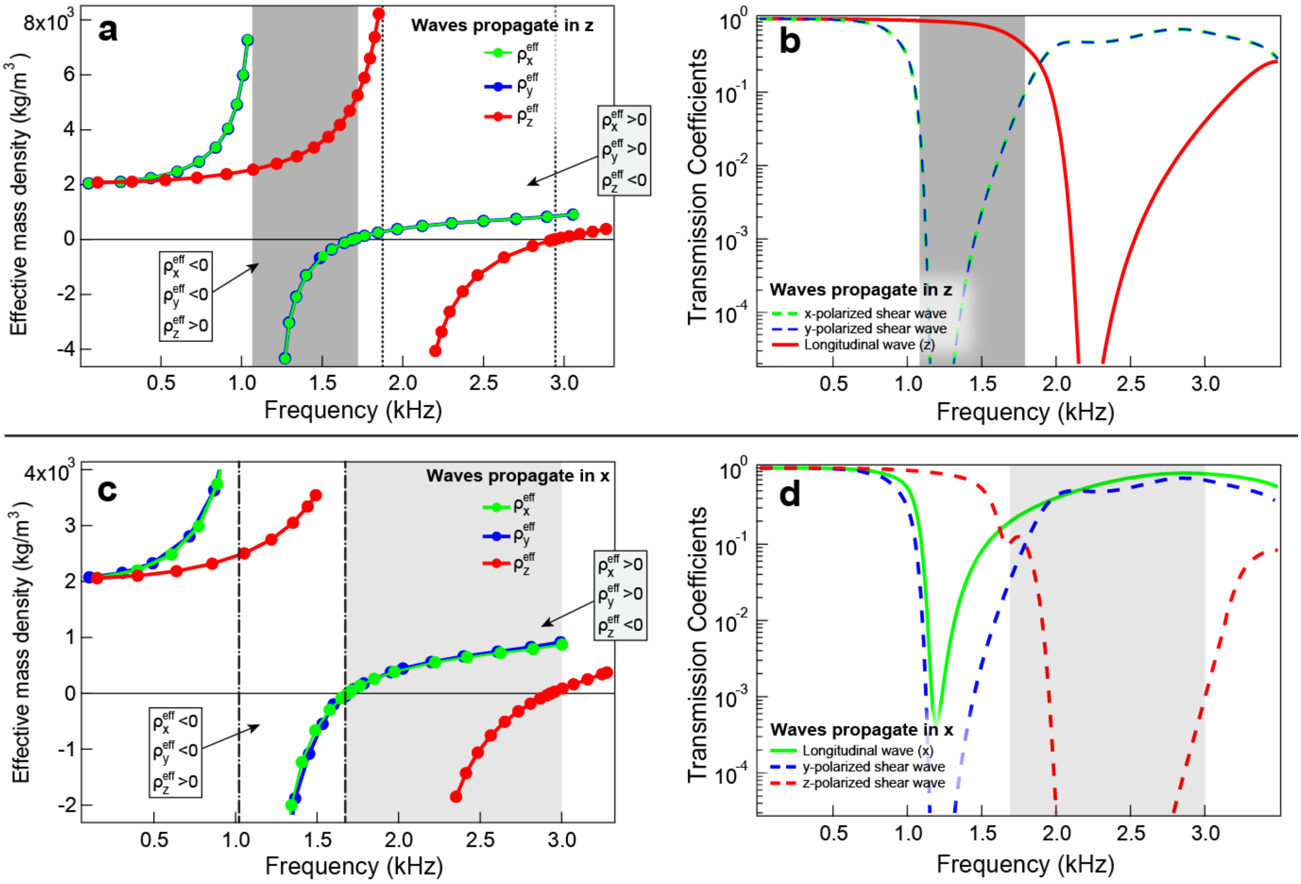

Supplementary Figure 5 Indefinite mass densities and wave transport properties of a three-dimensional lattice. For waves propagate in *z*-direction, the components of effective mass density tensor (ρ_i^{eff}) are shown in **a** as functions of frequency. Two regimes with indefinite mass density are shaded: $\rho_x^{eff} < 0$, $\rho_y^{eff} < 0$, $\rho_z^{eff} > 0$ in 1.1–1.7 kHz (dark grey); $\rho_x^{eff} > 0$, $\rho_y^{eff} > 0$, $\rho_z^{eff} < 0$ in 1.9–2.9 kHz (bounded by dotted lines). Simulated transmission coefficients are shown in **b**. Fluid-like elasticity, where only longitudinal waves can propagate, is found at 1.1–1.7 kHz (shaded dark grey). For waves propagate in *x*-direction, ρ_i^{eff} are shown in **c**; simulated transmission coefficients are shown in **d**. A partially fluid-like regime, in which longitudinal waves and *y*-polarized shear waves can propagate whereas *z*-polarized shear waves are forbidden, is found in ~1.7–2.9 kHz (shaded light grey in **c**, **d**). In this region, we have $\rho_x^{eff} > 0$, $\rho_y^{eff} > 0$, $\rho_z^{eff} < 0$. In **b**, **d**, solid/dashed curves represent longitudinal/shear waves, respectively. The curves' colors align with corresponding results in **a**, **b**. Note that ρ_i^{eff} in **a**, **c** were calculated with no dissipation in the materials, whereas dissipation presents in silicone rubber in the calculation of the transmission coefficients in **b**, **d**.

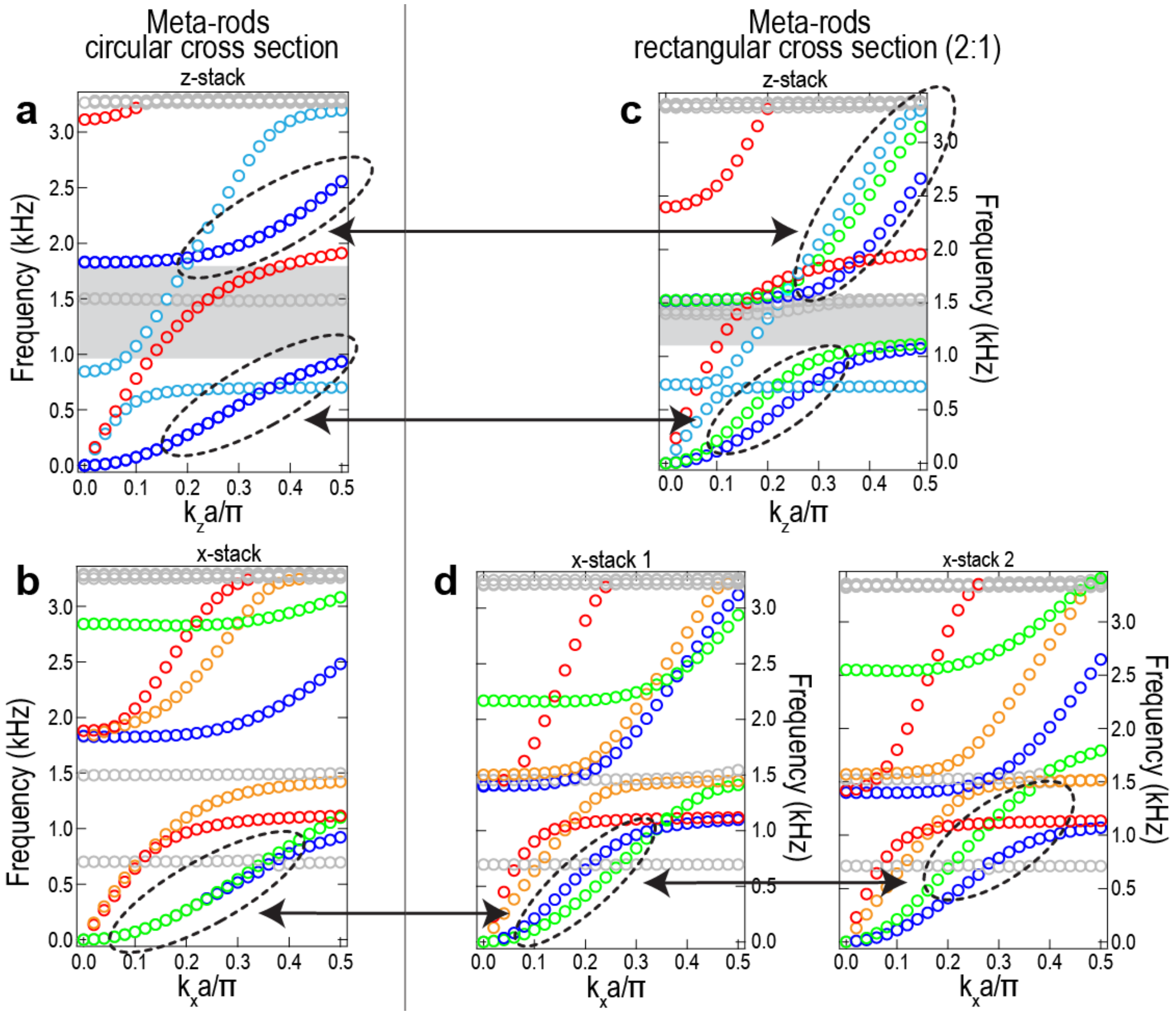

Supplementary Figure 6 Band structures of meta-rods with circular and rectangular cross sections. Meta-rods with circular cross section are shown in **a**, **b**; and meta-rods with rectangular cross section (aspect ratio 2:1) are shown in **c**, **d**. In all panels, red markers delineate branches associated with longitudinal modes; blue/green markers plot branches associated with flexural modes, and orange/cyan markers show torsional branches. Fluid-like elasticity can be found in both cases in *z*-stack meta-rods (grey shaded regions in **a**, **c**). The overall behaviors of the circular meta-rods are almost identical to the square meta-rods (the case studied in the main text). Rectangular meta-rods have additional anisotropy in their cross sections, consequently the two flexural branches split and have different propagation speed (enclosed by dashed ovals).

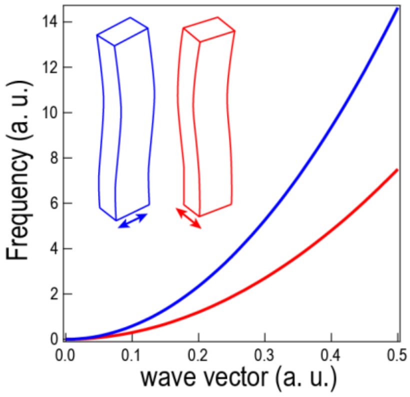

Supplementary Figure 7 For a rod with rectangular cross-section, the two flexural modes are no longer degenerate, but have different dispersions. If the rod the bending along the thicker direction (blue inset), the flexural modulus in effect is larger, thereby the wave speed is generally higher (blue curve). If the rod the bending along the thinner direction (red, inset), a smaller flexural modulus leads to lower wave speed (red curve). This observation will lead to interesting consequences for meta-rods as well (see Supplementary Fig. 6c, d).

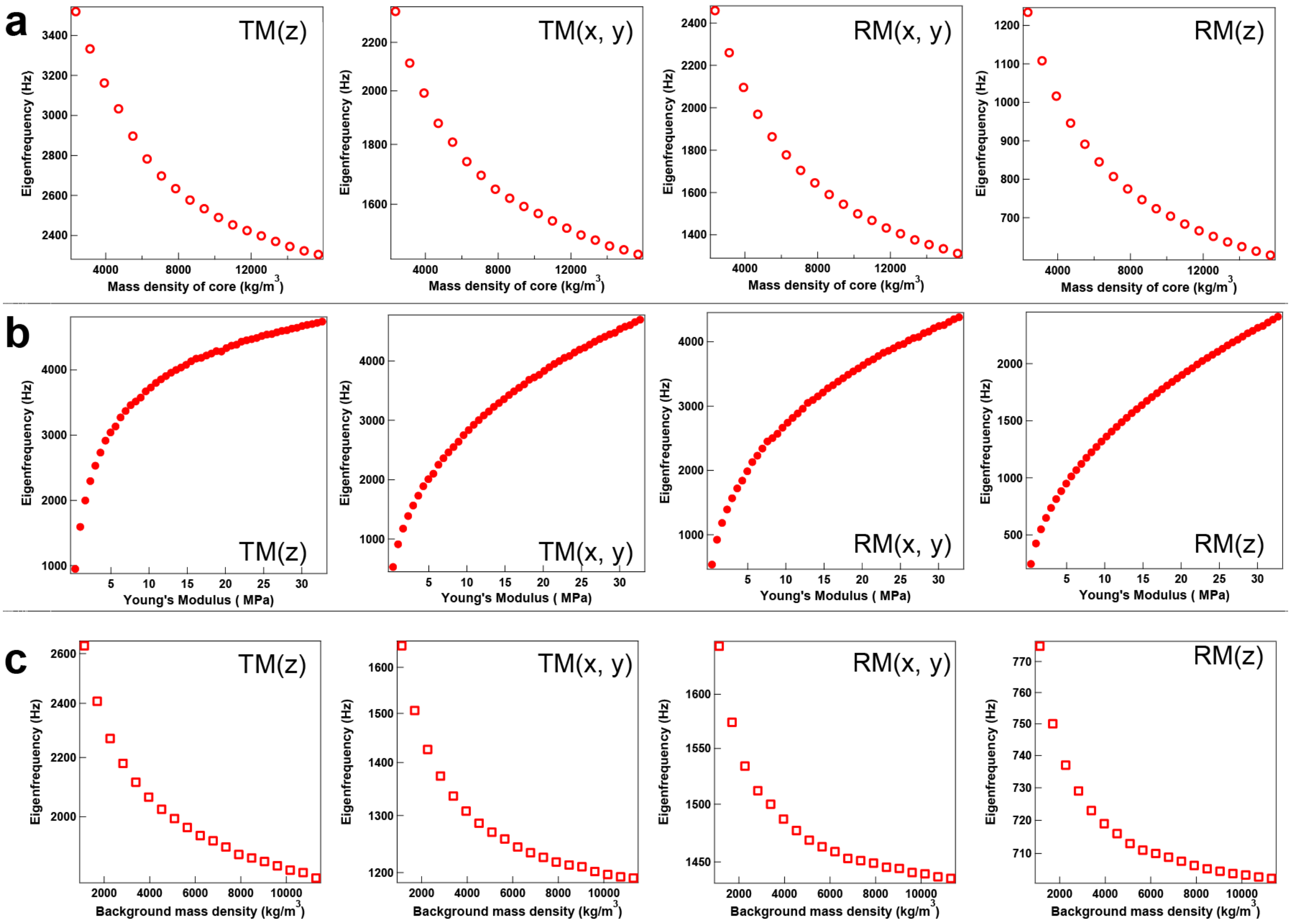

Supplementary Figure 8 Tuning the eigenfrequencies by material parameters. In **a**, the mass density of the cylindrical core is altered, whereas the silicone layer and epoxy maintain their properties. It is seen that all eigenfrequencies decrease as the core's mass density increases. In **b**, the Young's modulus of the silicone is

varied. Clearly, the harder (larger Young's modulus) the layer is, the higher the eigenfrequencies. Here, steel and epoxy are used as the materials of the core and cladding. In **c**, the mass density of the cladding (or background) is adjusted, whereas steel and silicone are used for the core and coating layer. Eigenfrequencies drop as the mass density increases.

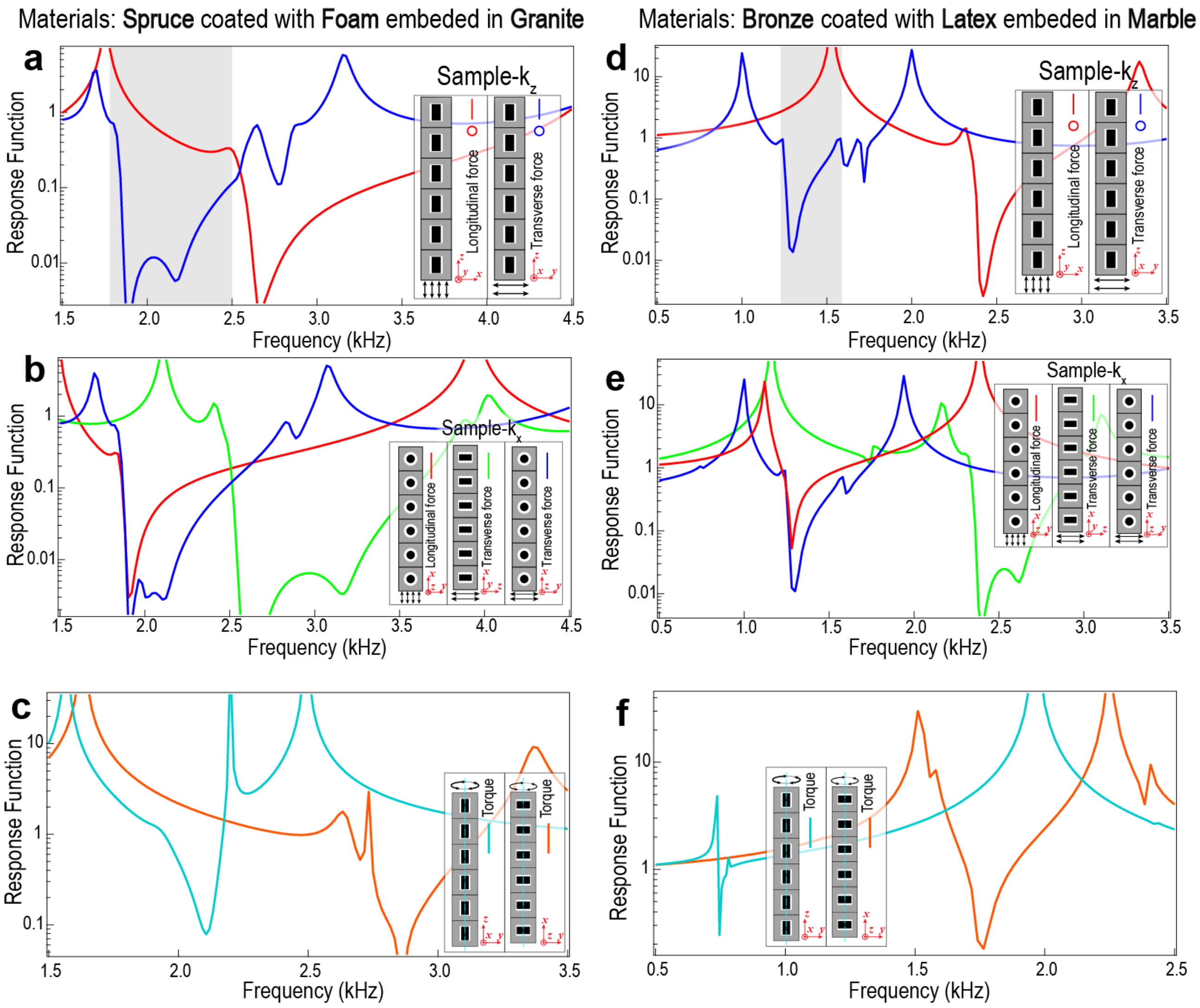

Supplementary Figure 9 Polarization bandgaps realized with alternative materials. Meta-rods constructed from unit cells made of a granite cylinder coated by foam embedded in spruce are shown in **a-c**. Fluid-like property can be found near 1.8–2.5 kHz (Sample-k_z, grey shaded region) and 1.8–2.4 kHz (Sample-k_x, partially fluid-like). Meta-rods constructed from unit cells made of a bronze cylinder coated with latex rubber embedded in marble are shown in **d-f**. Fluid-like property can be found near 1.2–1.6 kHz (Sample-k_z, grey shaded region) and 1.2–1.7 kHz (Sample-k_x, partially fluid-like). All curves are numerically calculated using COMSOL Multiphysics.

SUPPLEMENTARY NOTES

Supplementary Note 1. Tuning eigenfrequencies via silicone layer thickness

In the main text, we pointed out that eigenfrequencies of the translational modes (TMs) are tunable via changing the thickness of silicone rubber layer covering the steel cylinder core. This can be clearly seen in Supplementary Fig. 1, in which the eigenfrequencies are plotted against thickness of the silicone layer at different positions. Here, the eigenfrequencies are numerically calculated using COMSOL Multiphysics. Simply put, the eigenfrequency of TM(*z*) is sensitive to h_s (as defined in Supplementary Fig. 1a), i. e., the silicone thickness on the top and at the bottom of the steel cylinder (Supplementary Fig. 1b); whereas the eigenfrequencies of TM(*x*, *y*) are sensitive to r_s, i. e., the thickness of the silicone layer around the steel core's side surface (Supplementary Fig. 1c).

Supplementary Note 2. Transverse and longitudinal responses of sample-$\boldsymbol{k_x}$

A photographic image of sample-k_x is shown in Supplementary Fig. 2a (top). Sample-k_x is constructed by periodically repeating the unit cells along the *x*-axis. Therefore, the steel cylinders' axes are perpendicular to the rod's axis. Similarly, the sample is subjected to excitation from both transverse and longitudinal forces (experimental setups shown in Fig. 3a, b, main text). From the sample's geometry, it is easy to see that the longitudinal branch is excited together with the translational mode in *x*-direction (TM(*x*)), which has an eigenfrequency of ~1.6 kHz. A polaritonic dispersion with a bandgap is found at ~1.1–1.7 kHz (Supplementary Fig. 2a, red). This bandgap is observed in both experiment and simulation (Supplementary Fig. 2b, red markers and curve). The situation of flexural branches is slightly more complicated. Depending on the direction of the transverse force exerted, two situations can occur. When the transverse force is in *z*-direction, a *z*-polarized flexural branch and TM(*z*) are excited, giving rise to a flexural bandgap at ~1.4–2.6 kHz (Supplementary Fig. 2a & b, green

markers and curve). However, when the force is in *y*-direction, a *y*-polarized flexural branch together with TM(*y*) are excited. The flexural bandgap is then found at ~1.0–1.7 kHz (Supplementary Fig. 2a & b, blue markers and curve). From these observations, it is clear that the fluid-like property only exists for the *z*-polarized flexural waves in the regime of 1.7–2.6 kHz. Rotating the meta-rod by 90° along its axis (*x*-axis) will effectively turn off the fluid-like behavior, and yield a passband for both longitudinal and flexural waves in the same frequency regime (1.7–2.6 kHz).

Supplementary Note 3. Extraction of the effective moment of inertia

The moment of inertia is a wave parameter that is unique to rods[1]. The effective moment of inertia of the meta-rod per unit cell, $\overleftrightarrow{I}$, can be defined as $\Delta\vec{\tau} = \overleftrightarrow{I} \cdot \vec{\alpha}$. Here, $\Delta\vec{\tau}, \vec{\alpha}$ are the effective torque applied on the unit cell, and the effective angular acceleration of the unit cell, respectively. In the case of sample-k_x, we can easily calculate I_x, i. e., the effective moment of inertia per unit cell about *x*-axis (the rod's axis). The torque can be obtained from $\vec{\tau} = \int \vec{r} \times \vec{F} dydz$, where $\vec{F}$ is local force. For a unit cell, we find that $\tau_x = \int (y\sigma_{xz} - z\sigma_{xy}) dydz$ and $\Delta\tau_x = \tau_x|_{x=a} - \tau_x|_{x=0}$. Here, σ_{xz} and σ_{xy} are stresses. The angular acceleration can be obtained from $\vec{\alpha} = \int \vec{r} \times \frac{\vec{a}}{r^2} dydz$, where $\vec{a}$ is acceleration at each point on the yz surface. Thus, we have $\alpha_x = \int \frac{y a_z - z a_y}{y^2 + z^2} dydz / \int dydz$. The effective angular acceleration of the unit cell can be obtained as $\alpha_x = \frac{1}{2}(\alpha_x|_{x=a} + \alpha_x|_{x=0})$. By submitting α_x and $\Delta\tau_x$ into $\Delta\vec{\tau} = \overleftrightarrow{I} \cdot \vec{\alpha}$, we can obtain I_x.

Supplementary Note 4 Torsional response of sample-k_z

Torsional vibration can also be excited in sample-k_z by the setup shown in Fig. 3c (main text). From the unit cell's orientation, it is easy to see that the rotational mode about *z*-axis, i. e., RM(*z*), is excited. Mode profile of RM(*z*) is shown in Fig. 1c (main text). Naturally, RM(*z*) couples with the rod's

torsional vibration, giving rise to a polaritonic dispersion, together with a bandgap. The calculated band structure of sample-k_z is shown again in Supplementary Fig. 3a, with the torsional branch highlighted in cyan, and the bandgap shaded cyan. This bandgap is confirmed by finite element simulation of the response function, which found a response minimum near 0.75 kHz, as shown in Supplementary Fig. 3b. We have also calculated I_z, the effective moment of inertia per unit cell about *z*-axis. We found that $I_z < 0$ in 0.7–0.8 kHz (dashed curves in Supplementary Fig. 3b, right axis).

However, we are unable to observe the torsional bandgap of sample-k_z in experiments. This may be due to the following reasons. First, the bandgap itself is very small (<80 Hz, Supplementary Fig. 3a), making observation inherently difficult. Bandgap formation relies on the anti-crossing between a localized resonance and a propagating continuum. Generally, coupling strength determines the size of the bandgap. Here, the small bandgap indicates weak coupling. This brings us to the second possible reason. The weak coupling may owe to the microstructure of the metamaterial. Specifically, the steel cylinder has a curved side surface. In RM, the counter-rotating steel cylinder and epoxy rely on the compression and expansion of the silicone rubber to provide harmonic restoring torque. However, if the interface bonding is not ideal, slipping may occur between the steel/silicone and (or) silicone/epoxy interface. This is particularly likely when the interface is curvilinear, which is exactly the case for RM(*z*). Consequently, RM(*z*) may not be effectively excited even with rotational actuation, which results in the unsuccessful experimental attempts.

In contrast, for RM(*x*) and RM(*y*), the coupling strength must be larger, as indicated by the larger bandgap size (>200 Hz, orange shaded region in Fig. 2b, main text). This owes to the better structural restriction: for the steel cylinder to rotate about *x* or *y*-axis, there shall always be a certain degree of compression/expansion in the silicone rubber when relative rotational motions occur, since the side and ends of the steel cylinder meet in right angles. Therefore, the requirement on interface bonding is also far less stringent.

Supplementary Note 5. Numerical study of a three-dimensional array

By repeating the same metamaterial unit cells in all three spatial directions, a three-dimensional (3D) lattice can be constructed. Its band structure is shown in Supplementary Fig. 4. Here, *z* is the direction parallel to the steel cylinder's axis; and *x*, *y* are in-plane with the cylinder's ends. Likewise, the lattice is isotropic in *x* and *y*, and is anisotropic in *z*.

We make the following observations in Supplementary Fig. 4. First, only three branches are found in the long-wavelength limit, two of which are bulk shear waves (blue and green markers), the third one is a longitudinal wave (red makers). Both the longitudinal and the shear waves have linear dispersions. (In a bulk solid material, transverse branches are pure shear waves as opposed to flexural waves.) Second, rotational modes (RMs) still exist (grey markers). However, torsional branches disappear in this 3D lattice. Consequently all three RMs manifest mainly as flat bands, i. e., they couple minimally to stimulations. Some weak interaction with shear waves still exists[2], as shown near 1.5 kHz, $k_x a/\pi \sim 0.5$.

Third, owing to spectrally mismatched polarization bandgaps, fluid-like characteristic is found. For a wave incident in *z*-direction, bandgaps for both shear waves coincide, and overlap with longitudinal passband in the frequency regime of 1.1–1.7 kHz. This fluid-like region is shaded in dark grey in Supplementary Fig. 4. For incidence from *x*, however, shear waves can couple with TM(*z*) or TM(*y*), depending on the polarization. Fluid-like property exists only partially: *z*-polarized shear waves are forbidden in 1.7–2.9 kHz (light grey shaded in Supplementary Fig. 4), in which longitudinal waves and *y*-polarized shear waves can propagate. *Y*-polarized shear waves, however, have a bandgap that almost coincides with that of longitudinal waves.

The unique elastic properties of the 3D metamaterial can also emerge from an effective medium. Since all translational eigenmodes are clearly dipolar in their symmetry, they should dominantly induce anomaly in components of effective mass density tensor[3-5]. Due to the anisotropic unit structure, the effective mass density is also anisotropic. The effective mass density can be obtained from the Newton's second law[4,6], i.e.

$$\rho_i^{eff} = \frac{m_i^{eff}}{a^3} = \frac{F_i^{eff}}{\ddot{u}_i^{eff} a^3} = \frac{-F_i^{eff}}{\omega^2 u_i^{eff} a^3} \ , \qquad i = x, y, z.$$

Here ρ_i^{eff} is the effective mass density in the *i* direction; F_i^{eff} is the effective net force exerted on the unit cell in the *i* direction; u_i^{eff} is the effective displacement of the unit cell in the *i* direction, the double over-dots indicating second order time derivatives; and a is the lattice constant. F_i^{eff} and u_i^{eff} can both be obtained from the eigenfunctions.

We note that different eigenmodes should be chosen to obtain effective mass density in different directions. For waves propagating in k_z direction, we have

$$u_i^{eff} = \frac{\int u_i dxdy|_{z=a} + \int u_i dxdy|_{z=0}}{2a^2} \ ,$$

$$F_i^{eff} = \int T_{iz} dxdy\Big|_{z=a} - \int T_{iz} dxdy\Big|_{z=0} \ .$$

For waves propagating in k_x direction, we have

$$u_i^{eff} = \frac{\int u_i dydz|_{x=a} + \int u_i dydz|_{x=0}}{2a^2} \ ,$$

$$F_i^{eff} = \int T_{ix} dydz|_{x=a} - \int T_{ix} dydz|_{x=0} \ .$$

Here, T_{ij} are the components of the stress tensor, with $i, j = x, y, z$.

The effective mass densities obtained by eigenmodes in k_z are shown in Supplementary Fig. 5a. It is seen that in 1.1–1.7kHz, we have

$$\rho_x^{eff} < 0, \qquad \rho_y^{eff} < 0, \qquad \rho_z^{eff} > 0.$$

In electromagnetism, anisotropic effective parameters with different signs along different spatial directions are dubbed "*indefinite*"[7]. We therefore use the name "indefinite effective mass density". For waves propagating in *z*, ρ_z^{eff} affects longitudinal waves. With positive values, propagation of longitudinal waves in *z* is allowed in this frequency regime. On the other hand, both ρ_x^{eff} and ρ_y^{eff} are negative, which means shear waves are forbidden. Consequently, this frequency regime is the fluid-like regime, in alignment with the previous band structure analysis.

It is also seen that in 1.9–2.9 kHz,

$$\rho_x^{eff} > 0, \qquad \rho_y^{eff} > 0, \qquad \rho_z^{eff} < 0.$$

Namely, shear waves are allowed, whereas longitudinal waves are not.

We have further calculated the transmission coefficients for all three types of waves using COMSOL Multiphysics. The system parameters used here are identical to those shown in “Methods” following the main text. Dissipation is also added to silicone rubber. The results are shown in Supplementary Fig. 5b. We put six unit cells in the direction of incidence, whereas full periodicity are used for the four boundaries parallel to incidence. Excellent agreement with band structure analysis and effective medium prediction is achieved.

Effective mass densities of waves that propagate along k_x are shown in Supplementary Fig. 5c. Simply put, it is seen that $\rho_x^{eff} < 0$, $\rho_y^{eff} < 0$, $\rho_z^{eff} > 0$ in the frequency range of 1.1–1.7kHz. Therefore, longitudinal waves and *y*-polarized shear waves cannot propagate in this frequency regime. Only *z*-polarized shear waves are allowed. In ~1.7–2.9 kHz, we have $\rho_x^{eff} > 0$, $\rho_y^{eff} > 0$, $\rho_z^{eff} < 0$. Therefore, longitudinal and *y*-polarized shear waves are allowed, but *z*-polarized shear waves are forbidden. This regime is partially fluid-like. Likewise, simulated transmission coefficients confirm our analysis (Supplementary Fig. 5d).

In previous studies on acoustic metamaterials, the anisotropic mass density with both positive and negative components, i.e. indefinite mass density, have been shown to lead to hyperbolic dispersions[8-10], similar to electromagnetic metamaterials[11]. Such unique hyperbolic dispersion leads to important applications such as hyperlenses[10,12,13]. In the context of elastic waves, however, the indefinite mass density does not lead to hyperbolic dispersions with band gap in one direction and propagating states in the other direction. For simplicity, we let the modulus be positive and isotropic. If the *x* component of mass density is negative and the *y* component of mass density is positive, then longitudinal waves are forbidden in the *x* direction, but allowed in the *y* direction. Transverse waves, on the other hand, behave in the opposite way. Therefore, the bandgaps are polarization dependent, and there is no complete bandgap in both *x* and *y* direction[14]. This unique phenomenon is a direct consequence of the

richer freedoms of polarizations existing in elastic waves compared to acoustic and electromagnetic waves.

Supplementary Note 6. Geometry of meta-rod's cross section

Here, we investigate how the cross sectional shape affects the wave properties of the meta-rod. We keep all material parameters the same as those in the main text. The steel cylinder, silicone layer, and lattice constant also retain their geometric values. First, we note that both flexural modulus and torsional rigidity depend on the rod's cross sectional shape[1]. Therefore changing the cross sectional geometry will affect both quantities. For a torsional wave, a different torsional rigidity merely induces a different wave speed. However, for flexural waves the situation is slightly more interesting.

If a rod has cross-sectional shape of a circle, then it is considered isotropic in its cross-sectional plane. Flexural waves with both polarizations must still have the same dispersion relation and the same wave speed (at a given frequency). Consequently, overall behaviors of circular meta-rods can be almost identical to the square meta-rods. This can be seen in Supplementary Fig. 6a, b. In which all polarization bandgaps and the fluid-like region (grey shaded in Supplementary Fig. 6a) can be easily identified for both *z*-stack (in which the steel cylinders' axes are aligned and are parallel to the meta-rod) and *x*-stack (in which the steel cylinders' axes are perpendicular to the meta-rod) cases. Note that *z*-stack meta-rod is homogeneous in *xy*-plane, therefore its two flexural branches are degenerate (marked by black dashed ovals in Supplementary Fig. 6a).

If additional anisotropy is introduced, for instance, the rod's cross-sectional shape is changed into a rectangle, then flexural waves will pick up different dispersion relations for different polarizations. This is schematically explained in Supplementary Fig. 7 for a homogenous rectangular rod. If the rod is bending along the rectangle's long side (blue inset), the flexural modulus takes a larger value and the wave speed is generally higher (blue curve). In contrast, if the rod is bending along the rectangle's short side (red inset), the wave speed is lower (red curve). This observation also appears for meta-rods.

In Supplementary Fig. 6c, d, we plot the band structure of a meta-rod with a rectangular cross section with an aspect ratio of 2:1, i. e., the short side is $a = 60$ mm, and the long side is $b = 2a = 120$ mm. We clearly see that even for a z-stack meta-rod, the two flexural modes acquire different dispersions and split into two branches (Supplementary Fig. 6c, blue and green markers, further highlighted by black dashed ovals) – a clear indication of the different wave speeds for the two polarizations. Nevertheless, for this specific case, fluid-like elasticity still occurs (shaded grey in Supplementary Fig. 6c). Similar phenomenon can be seen in both x-stack cases (Supplementary Fig. 6d). Note that there exist two possible configurations for x-stack rectangular meta-rods: the steel cylinder can lie parallel to the short side (x-stack 1), or parallel to the long side (x-stack 2) of the cross section rectangle.

We also point out that torsional bandgaps are found in both circular and rectangular meta-rods, as shown in Supplementary Fig. 6 (cyan markers in a, c; orange markers in b, d). By changing the geometric shape of the cross section while maintaining all material parameters will inevitably cause changes of the total mass of the epoxy cladding, which will affect the spectral position and width of the polarization bandgaps. However, for the specific cases we are considering here, these changes are not substantial enough to affect the overall elastic properties.

Supplementary Note 7. Material choice

The properties of our metamaterial/meta-rod root in the local resonance of the subwavelength unit cell. This unit cell has a very simple physical model: a mass-spring-mass harmonic resonator wherein the eigenmodes along different spatial direction are spectrally detuned (Fig. 1a, inset). In general, this model is easily translated into a three-component composite wherein a rigid core is coated by relatively soft or elastic materials, and then is embedded in another relatively rigid material. Therefore, as long as the characteristics of these eigenmodes are not affected, we have a large degree of freedom in the choice of materials. However, choosing different materials will likely affect the eigenfrequencies. In a numerical study, we investigate how all the relevant eigenmodes change when we separately tune the

mass density of the cylindrical core (silicone and epoxy kept unchanged), the Young's modulus of the elastic layer (steel core and epoxy unchanged), and the mass density of the cladding material (steel core and silicone unchanged). All geometric parameters are identical to the unit cell in the main text. The results are shown in Supplementary Fig. 8. In short, increasing the mass density, or lowering Young's modulus leads to the reduction in the eigenfrequencies.

As a further demonstration, we numerically study the response functions of two specific examples using some widely available materials: granite coated by foam embedded in spruce, and bronze coated by latex rubber embedded in marble. We use the following material parameters (ρ is mass density, ν is Poisson's ratio, and E is Young's modulus): $\rho = 2800\ \mathrm{kg/m^3}$, $\nu = 0.25$, $E = 70\ \mathrm{GPa}$ for granite; $\rho = 120\ \mathrm{kg/m^3}$, $\nu = 0.33$, $E = 8 + 0.2i\ \mathrm{MPa}$ for foam; $\rho = 400\ \mathrm{kg/m^3}$, $\nu = 0.37$, $E = 11\ \mathrm{GPa}$ for spruce. And $\rho = 8700\ \mathrm{kg/m^3}$, $\nu = 0.34$, $E = 120\ \mathrm{GPa}$ for bronze; $\rho = 1300\ \mathrm{kg/m^3}$, $\nu = 0.48$, $E = 4 + 0.1i\ \mathrm{MPa}$ for latex rubber; $\rho = 2700\ \mathrm{kg/m^3}$, $\nu = 0.25$, $E = 40\ \mathrm{GPa}$ for marble. Geometric parameters are the same as in the main text: lattice constant $a = 60\ \mathrm{mm}$; cylinder core's height $h = 37.6\ \mathrm{mm}$ and radius $r = 15.8\ \mathrm{mm}$; silicone thickness is 1 mm on the top and at the bottom of the cylinder, and 5 mm around the side. Despite some of the material properties are vastly different from the steel/silicone/epoxy combination, it is clearly observed that fluid-like elasticity and torsional bandgaps persist, even though they are found at different frequencies as expected.

In short, the unique phenomena demonstrated in our work is not limited to the material combination shown in main text, but can be reproduced by a variety of material choices.

Supplementary References

1 Landau, L. & Lifshitz, E. *Theory of elasticity, course of theoretical physics*. Vol. 7 (Pergamon Press, 1986).

2 Peng, P., Mei, J. & Wu, Y. Lumped model for rotational modes in phononic crystals. *Phys. Rev. B* **86**, 134304 (2012).

3 Li, J. & Chan, C. T. Double-negative acoustic metamaterial. *Phys. Rev. E* **70**, 055602(R) (2004).
4 Lai, Y., Wu, Y., Sheng, P. & Zhang, Z.-Q. Hybrid elastic solids. *Nat. Mater.* **10**, 620-624 (2011).
5 Ma, G. & Sheng, P. Acoustic metamaterials: From local resonances to broad horizons. *Sci. Adv.* **2**, e1501595 (2016).
6 Wu, Y., Lai, Y. & Zhang, Z.-Q. Elastic metamaterials with simultaneously negative effective shear modulus and mass density. *Phys. Rev. Lett.* **107**, 105506 (2011).
7 Smith, D. R. & Schurig, D. Electromagnetic wave propagation in media with indefinite permittivity and permeability tensors. *Phys. Rev. Lett.* **90**, 077405 (2003).
8 García-Chocano, V. M., Christensen, J. & Sánchez-Dehesa, J. Negative refraction and energy funneling by hyperbolic materials: An experimental demonstration in acoustics. *Phys. Rev. Lett.* **112**, 144301 (2014).
9 Christensen, J. & de Abajo, F. J. G. Anisotropic metamaterials for full control of acoustic waves. *Phys. Rev. Lett.* **108**, 124301 (2012).
10 Shen, C., Xie, Y., Sui, N., Wang, W., Cummer, S. A. & Jing, Y. Broadband acoustic hyperbolic metamaterial. *Phys. Rev. Lett.* **115**, 254301 (2015).
11 Jacob, Z., Alekseyev, L. V. & Narimanov, E. Optical hyperlens: Far-field imaging beyond the diffraction limit. *Opt. Express* **14**, 8247-8256 (2006).
12 Li, J., Fok, L., Yin, X., Bartal, G. & Zhang, X. Experimental demonstration of an acoustic magnifying hyperlens. *Nat. Mater.* **8**, 931-934 (2009).
13 Lee, H., Oh, J. H., Seung, H. M., Cho, S. H. & Kim, Y. Y. Extreme stiffness hyperbolic elastic metamaterial for total transmission subwavelength imaging. *Sci. Rep.* **6** (2016).
14 Bückmann, T., Kadic, M., Schittny, R. & Wegener, M. Mechanical metamaterials with anisotropic and negative effective mass-density tensor made from one constituent material. *Phys. Statuts Solidi B* **252**, 1671-1674 (2015).
